# Application of Large Language Models in Medical Training Evaluation—Using ChatGPT as a Standardized Patient: Multimetric Assessment

**DOI:** 10.2196/59435

**Published:** 2025-01-01

**Authors:** Chenxu Wang, Shuhan Li, Nuoxi Lin, Xinyu Zhang, Ying Han, Xiandi Wang, Di Liu, Xiaomei Tan, Dan Pu, Kang Li, Guangwu Qian, Rong Yin

**Affiliations:** 1 West China Biomedical Big Data Center West China Hospital Sichuan University Chengdu China; 2 Department of Industrial Engineering Pittsburgh Institute Sichuan University Chengdu China; 3 Department of Medical Simulation Center West China Hospital Sichuan University Chengdu China; 4 Med-X Center for Informatics Sichuan University Chengdu China; 5 Department of Computer Science Pittsburgh Institute Sichuan University Chengdu China

**Keywords:** ChatGPT, artificial intelligence, standardized patient, health care, prompt engineering, accuracy, large language models, performance evaluation, medical training, inflammatory bowel disease

## Abstract

**Background:**

With the increasing interest in the application of large language models (LLMs) in the medical field, the feasibility of its potential use as a standardized patient in medical assessment is rarely evaluated. Specifically, we delved into the potential of using ChatGPT, a representative LLM, in transforming medical education by serving as a cost-effective alternative to standardized patients, specifically for history-taking tasks.

**Objective:**

The study aims to explore ChatGPT’s viability and performance as a standardized patient, using prompt engineering to refine its accuracy and use in medical assessments.

**Methods:**

A 2-phase experiment was conducted. The first phase assessed feasibility by simulating conversations about inflammatory bowel disease (IBD) across 3 quality groups (good, medium, and bad). Responses were categorized based on their relevance and accuracy. Each group consisted of 30 runs, with responses scored to determine whether they were related to the inquiries. For the second phase, we evaluated ChatGPT’s performance against specific criteria, focusing on its anthropomorphism, clinical accuracy, and adaptability. Adjustments were made to prompts based on ChatGPT’s response shortcomings, with a comparative analysis of ChatGPT’s performance between original and revised prompts. A total of 300 runs were conducted and compared against standard reference scores. Finally, the generalizability of the revised prompt was tested using other scripts for another 60 runs, together with the exploration of the impact of the used language on the performance of the chatbot.

**Results:**

The feasibility test confirmed ChatGPT’s ability to simulate a standardized patient effectively, differentiating among poor, medium, and good medical inquiries with varying degrees of accuracy. Score differences between the poor (74.7, SD 5.44) and medium (82.67, SD 5.30) inquiry groups (*P*<.001), between the poor and good (85, SD 3.27) inquiry groups (*P*<.001) were significant at a significance level (α) of .05, while the score differences between the medium and good inquiry groups were not statistically significant (*P*=.16). The revised prompt significantly improved ChatGPT’s realism, clinical accuracy, and adaptability, leading to a marked reduction in scoring discrepancies. The score accuracy of ChatGPT improved 4.926 times compared to unrevised prompts. The score difference percentage drops from 29.83% to 6.06%, with a drop in SD from 0.55 to 0.068. The performance of the chatbot on a separate script is acceptable with an average score difference percentage of 3.21%. Moreover, the performance differences between test groups using various language combinations were found to be insignificant.

**Conclusions:**

ChatGPT, as a representative LLM, is a viable tool for simulating standardized patients in medical assessments, with the potential to enhance medical training. By incorporating proper prompts, ChatGPT’s scoring accuracy and response realism significantly improved, approaching the feasibility of actual clinical use. Also, the influence of the adopted language is nonsignificant on the outcome of the chatbot.

## Introduction

### Background

In recent decades, large language models (LLMs) have experienced significant advancements [1,2]. LLMs are artificial intelligence (AI) models designed to comprehend and process natural human language, as well as generate it [1,3]. Concurrent with the progression of AI, LLMs have exhibited substantial potential in executing tasks involving natural language processing [4]. LLM applications range from article synthesis to summarization to patient diagnosis, illustrating their flexibility in providing valuable assistance [5-9]. Among the various types of LLMs such as the generalist language model, Flamingo, and Minerva [10], ChatGPT stands out as one of the critical milestone models, which is the primary focus of this study.

ChatGPT, developed by OpenAI, a US-based company, represents a significant advancement in language models [11,12]. This model facilitates user interaction with follow-up questions and is fine-tuned for controlled output [13,14]. Additionally, it can be integrated into custom applications via an application programming interface, allowing developers to craft chatbots and virtual assistants with tailored behavior [13]. Built on GPT architecture, ChatGPT currently comprises 2 primary variants, GPT-3.5 and GPT-4. A key characteristic of ChatGPT is that its output is significantly influenced by the input prompt and can be fine-tuned by modifying this prompt. Consequently, prompt engineering is essential for optimizing ChatGPT’s performance [15]. Despite ChatGPT and other LLMs, as well as the broader field of AI, being in developmental stages, they have demonstrated great potential in assisting human activities and may substitute human in certain tasks due to their low cost and high efficiency [16]. In the realm of medical education, traditional tests using standardized patients are no exception, involving substantial human labor and training costs [17-20].

The concept of a standardized patient, initially introduced by Barrows and Abrahamson [21], has become a widely recognized method in medical training and assessment [22,23]. In this context, “standardized” implies that the patient in a standardized patient scenario is trained to consistently portray a specific set of symptoms, medicine allergies, and medical history. Here, “patient” refers to an individual acting as if seeking medical care, unlike real patients, these are trained doctors or individuals simulating the diagnosis process to access medical professionals [23,24]. Consequently, a standardized patient is an individual trained to simulate a real patient, accurately portraying a set of symptoms or conditions. Medical students or doctors interact with standardized patients to practice and evaluate their clinical skills [22]. A critical aspect of this procedure is conversing with the standardized patient to obtain essential information for diagnosis, which is known as history-taking tasks. As the entire training process for an SP is both time-consuming and expensive, rural hospitals may even fail to provide such medical training due to poor access to high-quality health care services [25-27]. Our research aims to fill this research gap by applying LLMs, such as ChatGPT, in the assessment process involving standardized patients to tackle these problems. A critical factor influencing ChatGPT’s response is the prompt input given to the model [28,29]. Nevertheless, ChatGPT’s accuracy and precision require enhancement for medical applications [30,31]. Therefore, this study explored a novel approach to standardized patients in medical training evaluation by substituting human standardized patients with ChatGPT, as well as maintaining relatively high accuracy and precision. The study is conducted along 2 distinct dimensions. Initially, the capability and feasibility of ChatGPT functioning as a standardized patient are investigated. Subsequently, this study explores prompt engineering and revisions to enhance ChatGPT’s performance. Finally, this study examines the differences in scores and wording between original and revised prompt (RP) results generated by ChatGPT.

### Objectives

This study applied an exploratory approach to standardized patients in medical training evaluation by substituting human standardized patients with ChatGPT, as well as maintaining relatively reasonable accuracy and precision. The main objective can be divided into 2 stages of experiment. Initially, the capability and feasibility of ChatGPT functioning as a standardized patient are investigated. Subsequently, exploring prompt engineering and revisions to enhance ChatGPT’s performance, and also examining the differences in scores and wording between the original prompt (OP) and RP results generated by ChatGPT.

## Methods

### Standardized Patient Resource

The West China Medical Simulation Center of West China Hospital of Sichuan University has released a comprehensive standardized patient training script, encompassing a scenario-based conversational analysis and a specific performance criterion for participants, detailed in Table 1. This criterion emphasizes a procedure-oriented approach, where interactions with the standardized patient are critically evaluated and graded. The criterion comprises 2 principal aspects for evaluation: inquiry skills and humanistic care. The inquiry skills evaluation encompasses 4 dimensions, conversation arrangement, type of question, verifications, and use of professional jargon. In the domain of humanistic care, 2 dimensions are considered: speech and amiable behavior. Each dimension has 5 tiers, from tier 1 to tier 5, with ascending levels of performance. Comprehensive judgment criteria are illustrated in Table 1. ChatGPT exhibits limitations concerning criteria based on subjective judgment, as it lacks emotional response and is not fully adaptable to subjective grading. Therefore, this study focuses solely on the objective grading criteria. For example, an evaluation point could be whether the user inquired about the medicine history or not. Such criteria can be objectively assessed based on the conversation.

**Table 1:** Standardized patient consultation skills grading and scoring criteria. This scoring sheet can be used in standardized patient assessments to quantitatively evaluate the performance of candidates, where 5= best performance.

Skills		Ranking tiers				
		5-tier	4-tier	3-tier	2-tier	1-tier
**Inquiry skills**						
	Conversation arrangement	The beginning, middle, and end of the consultation are clear and precise, with questions asked in an orderly manner.	Between 5-point and 3-point	Most of the consultation is conducted in an orderly fashion, but the beginning and ending are not clearly defined.	Between 3-point and 1-point	The consultation lacks coherence and organization.
	Question types	Reasonable use of open-ended or closed-ended questions.	Between 5-point and 3-point	No open-ended questions, directly asking with closed-ended questions.	Between 3-point and 1-point	Frequently uses sequential and leading questions.
	Verifications	Conduct a comprehensive and thorough verification and reference.	Between 5-point and 3-point	The verification and reference are incomplete and not sufficient.	Between 3-point and 1-point	Did not conduct verification and reference.
	Professional jargon	The explanation is clear and easy to understand, not using complicated medical terminology.	Between 5-point and 3-point	The explanation is understandable, with minimal use of complex medical terminology.	Between 3-point and 1-point	Frequently uses complicate medical terminology.
**Humanistic care**						
	Speech	Appropriate speech speed and tone.	Between 5-point and 3-point	The speech speed and tone are mildly uncomfortable.	Between 3-point and 1-point	The speech speed and tone are noticeably uncomfortable.
	Amiable behavior	Appropriate response and comfort.	Between 5-point and 3-point	Provides responses and comfort.	Between 3-point and 1-point	No response or comfort.

### Study Design

To date, ChatGPT has introduced 2 versions of its language model: GPT-3.5 and GPT-4. OpenAI reports that GPT-4 exhibits improved word processing and memory retention capabilities. These advancements make GPT-4 particularly suitable for the aims of our research [32]. The preliminary step involves ascertaining the feasibility of ChatGPT functioning as a standardized patient in medical training evaluations. To gain preliminary insights, we conduct a preliminary, exploratory conversation with ChatGPT-4. We chose inflammatory bowel disease (IBD), a relatively rare condition with multiple symptoms that can be easily confused with other diseases [33,34]. In this experiment, the chatbot was given only the name of the disease, without detailed criteria, and was asked to perform as a standardized patient to assess users’ performance as if taking a standardized patient examination.

We asked a series of questions regarding IBD’s symptoms to determine if ChatGPT could respond appropriately like a real standardized patient. Subsequently, ChatGPT was requested to provide a score for our inquiry process, along with its criteria for assessment. The chatbot was evaluated using 3 different approaches: using poor, medium, and good inquiries. The criteria were as follows: in the poor inquiry approach, no questions pertinent to IBD were posed, and the language was unprofessional and overly casual. The medium inquiry approach exhibited a combination of relevant and irrelevant questions, with varying degrees of accuracy. Conversely, the good inquiry approach demonstrated both linguistic precision and a consistently accurate focus on relevant topics. Each level underwent 3 consecutive tests, and in each test, the chatbot generated the answer 10 times, from which an average score out of 30 (5 scores each × 6 groups) was calculated for each level.

For further exploration, a second phase of the experiment was designed. In this subsequent experiment, we used a detailed script accompanied by clear criteria provided by our clinical skill training center. An example from the script, featuring a perfect, full-score conversation between a standardized patient and a medical student, was used to test the chatbot. By modifying the dialog, including additions and deletions, the final score could vary. To initiate the conversation, the chatbot was provided with a patient’s medical record, along with a prompt outlined in Table 2. During the subsequent conversation, the questions on the script list were asked respectively to assess if the chatbot can extract information from the medical record precisely. Each answer was checked and compared to identify the flaws in responses, which were considered as key points for future improvement. At last, a score based on the given criteria was assigned by ChatGPT, along with a detailed analysis of the score. Repeated experiments (OP-1 to OP-5) were conducted 5 times, for each experiment, ChatGPT was asked to generate the score 30 times to obtain an average error rate for this overall preliminary experiment. From OP-1 to OP-5, the questions asked differed; therefore, the total standard score also varied. The questions asked and the total score were different, but the criterion was the same. From OP-1 to OP-5, the total score was in increasing order: 20/100, 60/100, 75/100, 85/100, and 90/100, respectively. A score of 20/100 is classified as “poor inquiry,” while scores of 60/100 and 75/100 are classified as “medium inquiry,” and 85/100 and 90/100 are classified as “good inquiry.” Given that the total score varied across each trial, we used a metric known as the score difference percentage (SDP) to quantify ChatGPT’s performance. This metric represents the error rate in terms of the percentage difference between expected and actual scores. The formula used is as follows.



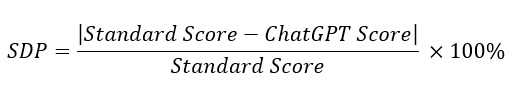



After the preliminary experiment was done, a full inspection of the entire conversation was given. ChatGPT’s performance on the following key points is focused on: the degree of anthropomorphism, clinical accuracy, and adaptability. Considering the degree of anthropomorphism, ensuring ChatGPT mimics a real patient accurately is crucial for its validity as a standardized patient, the chatbot should emulate the tone, behavior, and emotional responses typical of a real patient. In an authentic standardized patient assessment, to simulate a real-life patient, standardized patients may pose questions driven by anxiety, for instance, “Doctor, why does my stomach ailment keep recurring? Will it eventually lead to cancer?” [35]. It is acknowledged that ChatGPT may not initiate such inquiries due to its inherent limitations; however, we will investigate whether integrating a predesigned prompt enables the chatbot to generate these types of questions [36]. Moreover, the chatbot needs to communicate the symptoms effectively, maintaining a balance between being too vague and excessively detailed. It should provide information that is similar to what a real patient may disclose. For clinical accuracy, it is essential that the chatbot delivers precise information aligned with the provided medical record, which is a critical aspect of using ChatGPT in standardized patient assessments. The response from the chatbot must align with the corresponding description in the medical record. ChatGPT is expected to recall previous responses and consistently adhere to the documented medical history. For adaptability, in actual standardized patient tests, medical students may request information from the standardized patient that is not provided in the script or medical history; therefore, ChatGPT must be capable of inferring unspecified information. Following each evaluation, ChatGPT’s performance would be evaluated based on the earlier criteria, subsequently generating a list of issues. Based on the identified issues, the prompt was modified. Subsequently, a new series of experiments began, using RP. The final version of the prompt is determined when ChatGPT demonstrates satisfactory performance in terms of anthropomorphism, clinical accuracy, consistency in responses, and adaptability, among other factors. Additionally, the scores provided were closely inspected. We designed a comparative experiment involving the OP group. RP-1 to RP-5 were crafted correlating with OP-1 to OP-5. For each paired experiment, the questions posed and the total score were identical. The only difference between the OP and RP groups was the variation in the prompts provided. For each group, 30 runs were conducted to minimize the potential bias. Ultimately, the error rates will be compared with results from OPs to assess the efficacy of RP. A sample dialogue with ChatGPT is included in Multimedia Appendix 1.

Finally, the performance of the RPs was evaluated using a different standardized patient script to verify generalizability. In preliminary experiments, the dialogues with the chatbot were conducted in Chinese to minimize potential errors introduced by translation and the prompt provided was in English. However, according to a previous study on Chinese National Medical Licensing Examination questions, the translation of Chinese questions into English resulted in only a minor improvement (*P*=.16) [37]. To validate this research, the test group would be divided into 4 subgroups, representing all possible combinations of 2 languages used in prompts and dialogues, which were prompts in English (PE) + dialogue in English (DE); prompts in Chinese (PC) + dialogue in English (DE); prompts in English (PE) + dialogue in Chinese (DC); and prompts in Chinese + dialogue in Chinese (DC). All the tests in each group would be conducted 15 times to minimize the deviation caused by the inconsistency of the chatbot responses.

**Table 2:** Initial prompt given to ChatGPT^a^.

Prompt	Consideration
I'm a first-year medical student. Please help me with an interactive test case. Are you familiar with the procedure of a standardized patient assessment? Now, I want you to play the role of a standardized patient, while I play the role of the student. Follow these steps.	Initialization
Your basic situation: [The detailed medical history is provided to ChatGPT based the script]	Set up specific scenario
You need to answer the questions I'm asking you based on the facts.	Restrict the free response of ChatGPT
At the end of my consultation, you need to rate my consultation process, based on following criteria: [The specific scoring rules are provided]	Grade with detailed criteria

^a^This is the initial, unrevised prompt used for interactions with ChatGPT in the phase 2 study design, prior to prompt engineering.

### Data Analysis

The analysis software used in this research is R (version 4.3.3; R Foundation for Statistical Computing). For the first phase of the experiment, a 2-tailed *t* test is performed on the results to ascertain whether ChatGPT can differentiate between varying levels of problem relevance. In the second phase, the Shapiro-Wilk normality test was conducted to examine whether the data collected were normally distributed. For data not normally distributed, the Mann-Whitney *U* test was used to evaluate the significance of the differences in ChatGPT scores and standard scores, as well as between the OP and RP groups, thereby ascertaining the enhancements postprompt engineering. The data were collected during November 2023, December 2023, and August 2024.

## Results

### Feasibility Test

In the feasibility test, upon receiving the keyword “IBD,” ChatGPT automatically generated a virtual medical record, in subsequent interactions, it adhered to the information within the record, simulating a real patient’s behavior. For each inquiry quality group, 30 runs were conducted. Subsequently, ChatGPT provided an average score of 74.7/100 (SD 5.44) for a poor inquiry, an average score of 82.7 (SD 5.30) for a medium inquiry, and an average score of 85.0 (SD 3.27) for a good inquiry. These statistical data are presented in Table 3.

To demonstrate ChatGPT’s ability to distinguish the results, 2 inquiry model groups were established. The score differences between the poor and medium inquiry groups, between the medium and good inquiry groups, and between the poor and good groups were respectively examined. Both the score differences between the poor and medium inquiry groups (*P*<.001), between the poor and good groups (*P*<.001) were significant at a significance level of α=.05, while the score differences between the medium and good inquiry groups were not statistically significant (*P*=.16).

**Figure 1:**
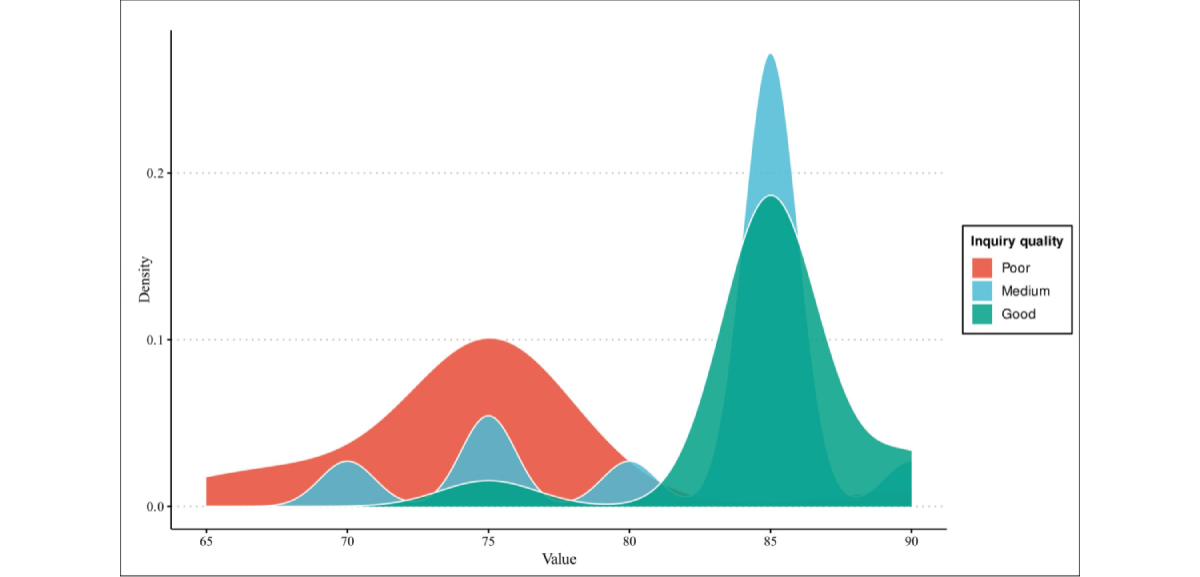
Scores given by ChatGPT in feasibility test. This figure illustrates the density distribution of scores given by ChatGPT when evaluating different levels of inquiry quality (poor, medium, and good) regarding IBD symptoms. Each quality level reflects varying degrees of relevance in questioning, with ChatGPT adhering to an IBD standardized patient scenario. The distribution shows how ChatGPT assessed each inquiry type. IBD: inflammatory bowel disease.

The density diagram of the scores is illustrated in Figure 1. Along with each score, the chatbot provided a detailed rating scale. Across various trials, ChatGPT’s criteria varied slightly, yet the core remained consistent: it consisted of 5 components: relativeness, inquiry into medical history, clinical reasoning, professionalism, and problem-solving.

**Table 3:** Scores given by ChatGPT in feasibility test^a^.

Inquiry quality	Mean (SD)
Poor	74.7 (5.44)
Medium	82.67 (5.30)
Good	85.00 (3.27)

^a^The table displays the mean (SD) scores provided by ChatGPT for different inquiry qualities related to inflammatory bowel disease symptom questions. The test involved three levels: “poor” (irrelevant questions and casual language), “medium” (mixed relevance), and “good” (precise and relevant). For each level, 30 interactions were conducted, and ChatGPT adhered to a virtual medical record generated for inflammatory bowel disease throughout the conversation, simulating a standardized patient.

### Performance Enhancement

**Figure 2:**
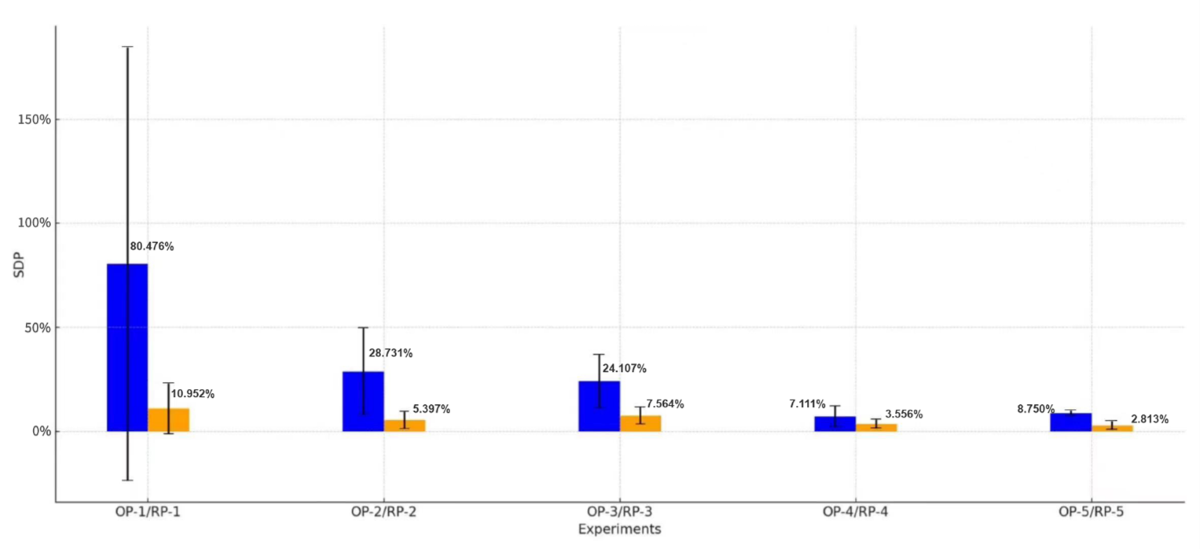
Error bar histogram of RP and OP experiments. This figure displays SDP across 5 experiments (OP-1 to OP-5 and RP-1 to RP-5) comparing OP and RP used for ChatGPT’s evaluation as a standardized patient. Each experiment involved 30 interactions, measuring the deviation of ChatGPT's provided scores from the standard scores. The blue bars represent the mean SDP for each OP group, and the orange bars indicate the corresponding RP group. Error bars denote the SD of SDP, highlighting variations in accuracy across prompt types. OP: original prompt; RP: revised prompt; SDP: score difference percentage.

The results from the preliminary experiment of the second phase demonstrated that ChatGPT can accurately replicate the clinical symptoms recorded in medical records; however, the scores it provided showed significant divergences compared to the standard scores. The results are presented in Figure 2. The upper band of each bar indicates the mean value of the SDP for each dataset, while the vertical line denotes SD. The data collected from the OP experiments are compared with the RP group subsequently.

**Figure 3:**
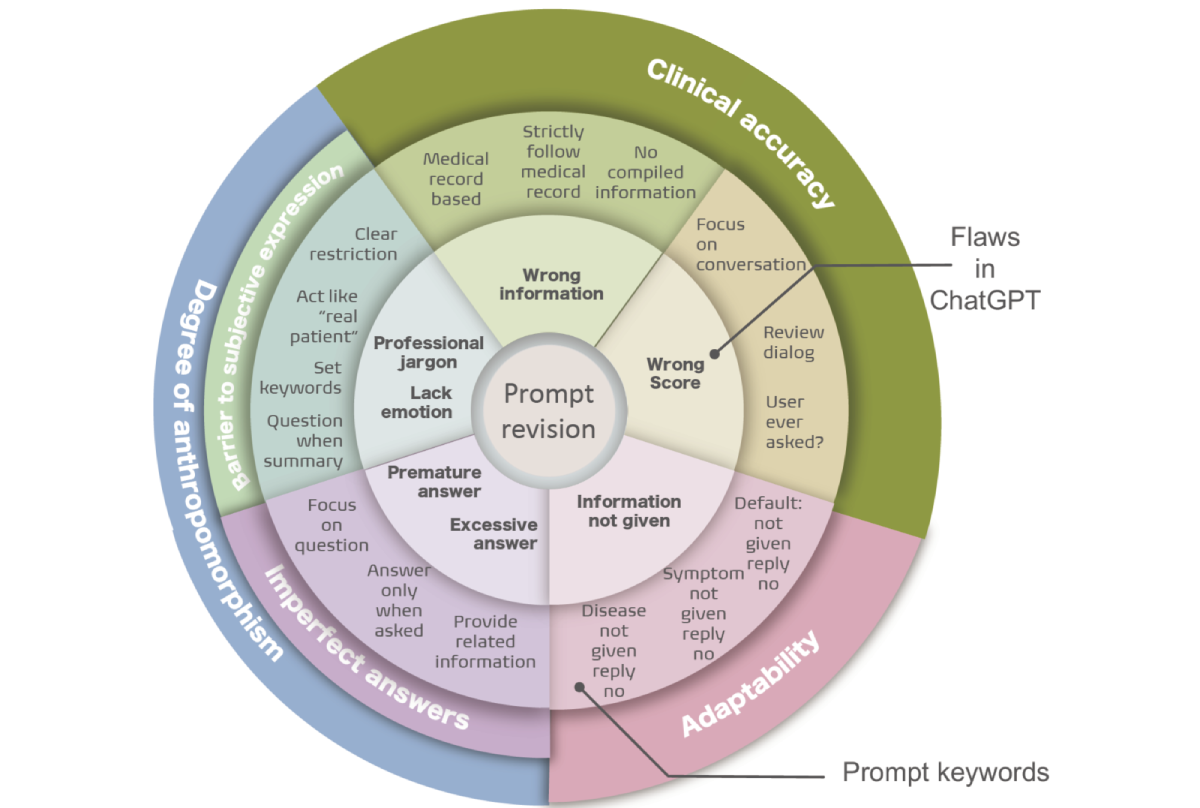
Prompt revision for ChatGPT in standardized patient assessment. This diagram summarizes key areas for prompt revision aimed at improving ChatGPT’s performance as a standardized patient. Focus areas include clinical accuracy, degree of anthropomorphism, and adaptability. Each segment outlines specific issues identified during initial tests—such as premature answers, use of professional jargon, and deviation from medical records—and corresponding prompt modifications. These adjustments were designed to enhance ChatGPT’s ability to simulate realistic patient behavior, adhere to medical records, and respond consistently during medical interactions.

To enhance performance, we focused on the following key aspects: degree of anthropomorphism, clinical accuracy, and adaptability. Regarding the degree of anthropomorphism, some responses provided by ChatGPT were notably overly professional with medical jargon. For instance, when inquired “Where do you feel unwell?” ChatGPT responded with “Processus xiphoideus.” This term is rarely used in everyday language, typically, one might say “My chest hurts.” Consequently, the chatbot was instructed to “Please perform like an ordinary person that does not have much professional knowledge in the medical field and avoid using professional jargons” to adjust its behavior. Moreover, without prompts, ChatGPT cannot replicate real-life standardized patients who may ask certain questions due to anxiety. To address this, the OP was enhanced with “When I present a summary of symptoms and seek your confirmation, please first respond to my inquiry.” Subsequently, simulate a patient tone characterized by anxiety, posing questions like “Can my illness be treated?” or “Is this a serious or minor illness?” Concerning clinical accuracy, several issues were identified. When queried, “Do you have any vomiting symptoms?” ChatGPT responded “Yes, basically undigested food. And my stools are normal, no black stools appeared” (this response originated from the provided medical history). The chatbot specifically inquired about the vomit habits; however, it responded with details on vomit habits and additionally mentioned the stool condition, which was documented in the medical record subsequent to the vomiting information. This indicates that ChatGPT may provide premature and overly comprehensive responses. A prompt can be introduced to address this issue: “Just answer the question each time I gave you, do not provide information that is not related to my question.” Furthermore, ChatGPT provided responses not aligning with the medical records. This issue, although infrequent, emerged several times during our experiment, when asked “Where are you feeling discomfort,” ChatGPT replied “head” instead of “stomach.” Although the chatbot can follow the content in medical records strictly in most situations, clinical accuracy is greatly favored, the chatbot must not make mistakes in this part. Additional prompts like “Please follow strictly with the information in medical record I provided you, do not compile information already provided in the medical record” can be added. When grading, ChatGPT sometimes did not focus on the conversation about standardized patients. After providing the criteria, the chatbot would just assume the conversation has happened. This could result in a high-scoring error rate. However, when initially adding the prompt “When scoring, please concentrate solely on the dialogue that took place during the standardized patient simulation. For each criterion listed, review our conversation history to determine whether I asked the specified question. If I did not, please assign a zero for that criterion.” Regarding adaptability, when ChatGPT was asked about diseases or symptoms not provided in the medical record, the chatbot was unable to answer these questions accurately, while the correct response should be “No.” For instance, when being asked “Have you experienced a heart attack,” ChatGPT responded with “Sorry, I cannot confirm this, as it is not indicated in the medical record.” As a medical record serves as a checklist for the patients’ disease history and symptoms experienced to date, it should be a complete reflection of the patients’ medical situation. Therefore, when asked about the symptoms or disease that is not provided in the medical record, ChatGPT’s answer should be “No, I do not have this symptom or disease.” Additional prompt can be added to ascertain the adaptability: “The patient’s all disease history and symptoms are as described in the medical record. The patient does not have any disease or symptoms not mentioned.” Our results are shown in the circular diagram in Figure 3. Combining all the revisional prompts, new prompts are listed in Table 4.

The results of the RP group are shown in Figure 2. We first used the Shapiro-Wilk normality test to determine whether the data in each experiment were normally distributed. The results show that all the data we got, both in OP and RP groups, were not normally distributed (all *P*<.001). Then Mann-Whitney *U* test was used to determine the significance between the mean of each dataset and the standard score. For instance, considering the OP-1 group, the standard score is 20/100, we used the Mann-Whitney *U* test to test if the difference between the mean of real scores ChatGPT provided in OP-1 and number of 60 has significance. Then, Mann-Whitney *U* tests were conducted between each paired OP and RP groups, respectively, to determine if RP indeed improved the performance of ChatGPT. The detailed result is shown in Table 5.

**Figure 4:**
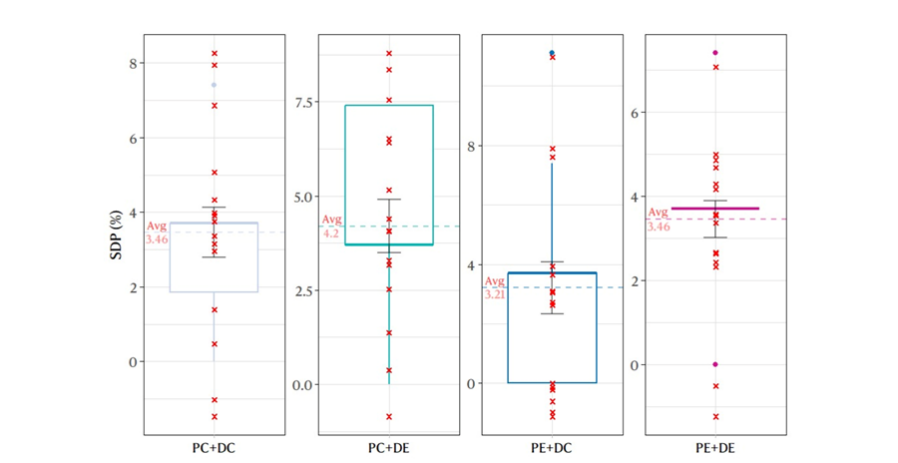
Experiment results using new script. This figure displays the performance of ChatGPT using revised prompts on another script across 4 language combinations: PC+DC, PC+DE, PE+DC, and PE+DE. Each group underwent 15 trials, and the results were analyzed based on the SDP metric, which measures the accuracy of ChatGPT’s scoring. The Mann-Whitney U tests indicated no significant differences between any group pairs, suggesting similar performance across language combinations. PC: prompts in Chinese; PE: prompts in English, DC: dialogue in Chinese; DE: dialogue in English. SDP: score difference percentage.

Using another new patient script, a total of 60 experiments were conducted on 4 test groups. The results are shown in Figure 4. The PE+DC group exhibited the highest performance, achieving an average SDP value of 3.21%. The PC+DE group demonstrated the lowest performance, recording an average SDP value of 4.2%. The average SDP values achieved by the PC+DC and PE+DE groups were generally identical (SDP=3.46%). However, the PE+DE group showed superior performance over the PC+DC group, as evidenced by a smaller deviation in results. Meanwhile, the results from the Mann-Whitney *U* tests, conducted across all groups, indicated no significant differences (*P*=.65, between PC+DC and PE+DC; *P*=.46, between PC+DC and PC+DE; *P*=.98, between PC+DC and PE+DE; *P*=.29, between PE+DC and PC+DE; *P*=.51 between PE+DC and PE+DE; and *P*=.37, between PC+DE and PE+DE).

**Table 4:** The revised prompt given to ChatGPT^a^.

Prompt	Consideration
I'm a first-year medical student. Please help me with an interactive test case. Are you familiar with the procedure of a standardized patient assessment? Now, I want you to play the role of a standardized patient, while I play the role of the student. Follow these steps:	Initialization
Your basic situation: [The detailed medical history is provided to ChatGPT based the script]	Set up specific scenario
You need to answer the questions I'm asking you based on the facts.	Restrict the free response of ChatGPT
Please perform like an ordinary person that does not have much professional knowledge in medical field. Avoid using professional jargons.	Avoid professional jargon
When I present a summary of symptoms and seek your confirmation, please first respond to my inquiry. Subsequently, simulate a patient tone characterized by anxiety, posing questions like 'Can my illness be cured?' or 'Is this a serious or minor illness?'	Set up question due to anxiety
Just answer the question each time I gave you, do not provide information that is not related to my questions.	Avoid premature and excessive answer
Please follow strictly with the information in medical record I provided you, do not compile information that is already provided in medical record.	Avoid wrong information
When giving score, please only focus on the conversation happened in standardized patient simulation, for each list in criteria, review our conversation history to check if I have ever asked this question, if not, then you give me a zero for this list.	Improve grading accuracy
The patient’s all disease history and symptoms are as described in medical record. The patient does not have any disease or symptoms not mentioned.	Improve adaptability
At the end of my consultation, you need to rate my consultation process, the specific scoring rules are as follows: [The specific scoring rules are provided]	Grade with detailed criteria

^a^This is the revised prompt used for interactions with ChatGPT in the phase 2 study design, after prompt engineering.

**Table 5:** Statistical data for experiment, using SDP^a,b^.

Experiment order	Real score			
	Mean (SD), %	Mean ChatGPT score	Accurate score	*P* value
OP^c^-1	80.476 (1.058)	12.633	7	<.001
RP^d^-1	10.952 (0.123)	7.033	7	.28
OP-2	28.731 (0.212)	26.567	21	<.001
RP-2	5.397 (0.041)	20.333	21	<.001
OP-3	24.103 (0.131)	32	26	<.001
RP-3	7.564 (0.039)	24.033	26	<.001
OP-4	7.111 (0.051)	32.134	30	<.001
RP-4	3.556 (0.021)	29.8	30	.81
OP-5	8.750 (0.013)	34.8	32	<.001
RP-5	2.813 (0.021)	32.833	32	<.001
OP-overall	29.834 (0.550)	—^e^	—	—
RP-overall	6.056 (0.068)	—	—	—

^a^SDP: score difference percentage.

^b^The table presents the accuracy of ChatGPT’s scoring during standardized patient assessments, comparing the OPs and RPs. The scoring measures how well ChatGPT’s responses align with predefined standard scores for each scenario. Metrics include the mean and SD of the SDP, indicating the deviation between ChatGPT’s scores and the expected standards. The table also provides accurate scores and *P* values from Mann-Whitney *U* tests, evaluating the significance of differences between ChatGPT’s scoring and the expected results.

^c^OP: original prompt.

^d^RP: revised prompt.

^e^No data.

## Discussion

### Principal Findings

#### Phase 1: Preliminary Experiment

In this study, we conducted an exploratory study to examine the potential of using ChatGPT as standardized patients in medical education. Also, the performance enhancement after RP was evaluated. The knowledge of ChatGPT is not updated every day, therefore the precision and accuracy of the chatbot may decrease when it comes to new diseases and medicine [30,38]. All the papers designed in this study for ChatGPT evaluation do not include diseases newly found, as all the analyses and questions provided to ChatGPT were conducted between November 2023 to December 2023. We found that with refined prompts, ChatGPT could simulate patient interactions effectively, demonstrating improvements in realism, clinical accuracy, and adaptability. The use of ChatGPT as a standardized patient offers a cost-effective alternative to traditional human standardized patients, potentially enhancing access to medical training, especially in resource-limited settings.

Our results suggest that, in the preliminary experiment, poor inquiry is most dense in a relatively high score of around 75, this score seems to be higher than expected as all 4 questions inputted to ChatGPT are basically irrelevant to the standardized patient test. However, ChatGPT is an LLM that is finetuned by humans, it is finetuned to minimize offense to humans [39,40] It is understandable that ChatGPT tends to give the user a higher score even if all the questions are wrong. As to the chatbot, it may seem to be offensive if providing a low score to human users while not having a detailed criterion. This problem disappeared in the later experiments where detailed criterion was provided. As for the medium inquiry way, ChatGPT provided an average score higher than that of a poor inquiry model. Our results suggest that the score of the medium inquiry group is the most spread, as well as the largest data range among the 3 models. This reflects that in the field of standardized patient assessment, ChatGPT can distinguish between a relevant question and an irrelevant one, but its performance is not stable and easily affected without proper definition and restriction.

Even though the average score difference between medium and good inquiries is not significant, good inquiry has the smallest variance with the highest data density. As our results suggest, the difference between poor and medium groups is significant, while the difference between medium and good is not statistically significant. Considering other trends, however, the increase in the average score, along with other data features, with respect to the increase and difference in inquiry question relevance, indicate that ChatGPT is aware of which question does not belong to a standardized patient assessment process and which question belongs to it. Although it cannot distinguish clearly without a concrete prompt, the chatbot is familiar with the process of standardized patient assessment. However, its criterion is different from the criterion provided by experts, and the scores it provides are erroneous. This is sufficient to provide evidence for the feasibility of using ChatGPT to perform standardized patient training.

#### Phase 2: Prompt Engineering

The preliminary experiment of the performance enhancement set up a blueprint of the whole experiment. As illustrated in Figure 2, in the first few experiments, the improvement of RP is quite significant. For instance, in the OP-1 experiment, the mean score has the SDP around 0.8 which means that the score provided by ChatGPT is 1.8 times higher than the accurate score. With an overall average SDP of 29.834%, this shows that ChatGPT’s behavior is highly unpredictable and unreasonable, and thus not feasible for application based on the current method. One noticeable trend from Figure 2 is that, when the experiment continues, the SD and average score become more accurate. This is not because of the fact that the performance of ChatGPT improves as the experiment is conducted, rather, literature suggests ChatGPT falls short when handling similar tasks. Without prompt limitation, the chatbot tends to include the content of the whole conversation into grading, beyond the user’s query. For example, if the user asked 1 question that addressed 1 key point in the criteria, but ChatGPT’s reply went beyond the scope of this question, its answer may cover two or more key points in the criteria. Therefore, even though the user did not explicitly inquire about certain key points, ChatGPT’s expansive response might still count these unasked points during grading. Consequently, this feature will let ChatGPT tend to provide a higher overall score.

To ensure a stable, accurate performance in standardized patient assessment, prompt engineering plays a critical role. ChatGPT’s output can be generally modified and controlled by changing prompts, thus a good prompt with many restrictions can constrain ChatGPT to guide the chatbot to our ideal output. The enhancement and revision of prompts become pivotal, which is the most significant and pivotal contribution of this study. The reasons why choosing the degree of anthropomorphism, clinical accuracy, and adaptability as our evaluation key point is based on ChatGPT’s prompt optimizing key features [41,42]. In the context of standardized patient training, the chatbot is required to perform like a real person, moreover, it must adhere more closely to the behaviors of a real-life patient, which is the essence of the test. In achieving so, the fundamental requirement for the chatbot is to have a high level of anthropomorphism which consists of 2 major problems discovered in this research: barrier to subjective expression and imperfect answers. For the former aspect, ChatGPT tends to use professional medical jargon to illustrate symptoms in medical reports which do not accurately reflect its role as a real patient. Also, in only 1 of 5 experiments, ChatGPT responded to a question as if it were experiencing anxiety. In OP-3, when asked “Have you ever been to the hospital before for this disease?” ChatGPT first answered the question objectively, then subjectively added, “I fear that this disease has lasted for too long, so I came here to consult you.” Although the case that ChatGPT has an anxiety tone is rare, this demonstrates that the chatbot can imitate this tone, requiring only some constraints in the prompt. Future studies may explore the possible prompts engineering to improve its performance in simulating human emotions. In essence, the performance of ChatGPT in the preliminary experiment is unstable and poor; however, it possesses the capability for preliminary testing yet lacks guidance, thereby necessitating optimization via prompt engineering [43], underscoring the significance and necessity of the second-phase experiment.

#### Ultimate Performance Enhancement

After applying RP, ChatGPT used descriptive words to answer questions like “Where is your discomfort,” instead of detailed medical jargon, and the chatbot asked questions like “Why my symptoms persist?” “Can my disease be cured?” After the user has summarized the conversation. Though the chatbot’s tone may seem “mechanical,” it indicates that RP indeed works. For the latter aspect, the imperfect answers here refer to the chatbot’s premature and excessive answers, which significantly influence grading. This part has a great influence on grading. For instance, when asked “Have you ever had other diseases in stomach?” the chatbot first answered yes, and explained when diagnosed and with what diseases. After providing sufficient information for the question, ChatGPT added “I have been to the hospital for the first time for this 2 months ago.” The answer may seem reasonable. Yet, during grading, ChatGPT automatically awarded full marks for “Asking about information on previous hospital visits,” justifying that the user had inquired about it. However, no question related to this was asked in that conversation. When asked for more detailed information, ChatGPT responded that since the content of the conversation mentioned the patient had gone to the hospital 2 months ago, it should count; even though this information was provided by the chatbot subjectively, not in response to the user’s question. Therefore, premature and excessive questions not only influence degrees of anthropomorphism but also contribute dramatically to grading errors. Subsequently, clinical accuracy is also a concern [44]. When restrictive prompts such as “Focus on my question” and “Answer only what is asked” are used, ChatGPT began to use plain, short answers to respond, from one extreme to another, but the answer contains the essence without premature questions, making these responses preferable. During the preliminary experiments, ChatGPT can strictly follow the medical record, only once mistaking a stomachache for a headache. Additionally, in 1 conversation, the chatbot provided compiled information on the medical history. Therefore, to assure accuracy, a restrictive prompt asking ChatGPT to base answers only on the record is crucial. Also, the chatbot must be accurate when giving the score, as this is the only quantitative reflection of ChatGPT’s performance in correctly evaluating medical students’ performance who are taking the standardized patient examination.

With RP, the chatbot now focuses on the content that appears in the standardized patient training conversation, before that, it sometimes mistook the content of the criteria as part of the judgment, resulting in full mark. Adaptability is also important, RP made a ChatGPT response with “No” for the symptoms asked but not in medical history in the following experiment. After applying RP, ChatGPT’s performance improves dramatically. One of the most serious problems in using ChatGPT is that the answer it provides has a high level of randomness and this is hard to avoid. The average SDP improved to 20 times higher than OP results. Considering the high randomness in answers provided by the chatbot, this result is quite acceptable. Also, the score deviation for each experiment is quite small, the average SD decreased to 0.068 from 0.55 in the original group results. By adding these prompts, we reduce SD to nearly one-fifteenth of its original value. Massive differences in average value and SD indicate that by giving our extra, RP, the performance of ChatGPT as a standardized patient improves significantly. The significant difference between the OP and RP results indicates that RP improved ChatGPT’s performance significantly. RP-2, RP-3, and RP-5 generally have lower SD with a lower mean SDP, which represents a higher accuracy and precision than their corresponding OP groups. To fully use ChatGPT in a standardized patient training process, good accuracy and consistency in responses are required. After applying our RP, ChatGPT has an overall SDP of 6.056%. This score is closer to 0% and improves a lot compared to the un-RP group (29.834%), even though there still remains space to improve. The results also indicate that ChatGPT’s performance can be fine-tuned through the revision of prompts provided to it. Future studies may conduct a more comprehensive examination to reduce the SDP further.

The efficacy of RP was confirmed through its universal applicability when tested on another script, achieving a similar average SDP to our preliminary script. Furthermore, validation tests conducted on the new script also indicated that the impact of language on chatbot performance is insignificant (*P>.*05, for all groups). The observed performance deviation is within acceptable limits. However, the highest accuracy rate was observed in the test group that used English prompts and Chinese dialogues.

### Limitations

There are several limitations in this study. First, to minimize errors associated with word translation, we conducted the simulated standardized patient test dialog with ChatGPT in standard simplified Chinese [45]. Nevertheless, the prompt provided to ChatGPT was in English to ensure accuracy. Although a separate experiment was conducted to confirm the minor impact of language used on the performance of ChatGPT, future studies may test the performance of ChatGPT on standardized patients in other languages and corresponding prompt engineering to improve its performance. Secondly, the criteria provided to ChatGPT involved only objective key points. In real standardized patient tests, some subjective judgments may also be made accordingly. Finally, the prompt revision we provided represents the optimal approach. Further research is necessary to use ChatGPT more effectively in standardized patient training.

### Conclusions

ChatGPT, as a representative LLM, has much potential application in medical assessment. Our study suggests that it is feasible to use ChatGPT as a simulated patient for standardized patient evaluation and scoring. The output performance concerns introduced by the randomness of ChatGPT could be improved by adding detailed, restrictive prompts. By using prompts revised in this study, the accuracy of ChatGPT’s scoring significantly improved to an acceptable level that could be used in actual standardized patients involved in medical training. Additionally, ChatGPT’s responses during the conversation became more accurate and lifelike. It is worth noting that, in this application scenario, the influence of the adopted language on the chatbot’s outcome is nonsignificant. Overall, ChatGPT’s accuracy and performance in standardized patient assessment are acceptable, but they highlight the need for continuing improvement before it can be used as a fully trustworthy clinical assessment method.

## Appendix

Multimedia Appendix 1 A sample interaction dialogue with ChatGPT for RP-2 (revised prompts) group (standard score=21).

## Abbreviations

AI: artificial intelligence
DC: dialogue in Chinese
DE: dialogue in English
IBD: inflammatory bowel disease
LLM: large language model
OP: original prompt
PC: prompts in Chinese
PE: prompts in English
RP: revised prompt
SDP: score difference percentage

## Data Availability

The datasets generated during and/or analyzed during this study are not publicly available due to institutional case privacy and a large number of interaction dialogs but are available from the corresponding author on reasonable request. Other than the sample dialogue provided in the Multimedia Appendix 1, additional detailed dialogues with ChatGPT may be available by contacting the corresponding author.
