# Supplementary material for: Application of Large Language Models in Medical Training Evaluation—Using ChatGPT as a Standardized Patient: Multimetric Assessment
Source: J Med Internet Res. 2025 Jan 1;27:e59435. doi: 10.2196/59435 (PMC11736217; doi:10.2196/59435)
Supplement: Multimedia Appendix 1 [file jmir_v27i1e59435_app1.docx]

**Table S1.** A sample interaction dialogue with ChatGPT for RP-2 (revised prompts) group (standard score=21).

| ***ChatGPT*** | ***User*** |
| --- | --- |
|  | I'm a first-year medical student. Please help me with an interactive test case. Are you familiar with the procedure of a Standardized Patient assessment? Now, I want you to play the role of a standardized patient, while I play the role of the student. Follow these steps:  Your basic situation: The patient, Zhang Hong, male, 38 years old, associate researcher from Hangzhou, Zhejiang Province, came to the outpatient clinic on 8 March 1995 because of recurrent epigastric pain for 4 years, aggravated for 3 months. Four years ago, due to dietary disorders, work and feel epigastric pain, for the subxiphoid burning pain or dull pain is not accompanied by acid reflux, ergonomics, pain pulling to the vest, mostly after meals or at night, can still be tolerated, each time after eating or hot drinks to alleviate the episodes continue for about half a month to get better, untreated. Since then, there have been 4-5 similar episodes per year with the same cause, which improved after rest and dietary moderation (e.g., soft food and light diet). Two years ago, she visited our hospital and was asked to take gastroscopy, but she did not do so. She took 3 tablets of GastroSuprine 6-7 times a day and got better, and then stopped taking the medicine after 1 month. In March, due to heavy scientific research tasks, often staying up late, irregular life, epigastric pain reoccurred, accompanied by nausea, vomiting celiac disease 2 times, with the smell of food, large amount. After the outpatient clinic of the First Municipal Hospital, the patient used 1 packet of Deluxe Punch for 4 times a day, which slightly improved the pain, but the symptoms still existed, and she came to the clinic because she was concerned about the deterioration of the lesion, whether there was a tumor, and whether she should undergo an examination and surgery. After 4 years of illness, she lost 5kg of body weight, her appetite decreased from 400g to 200g per day, and she was weak and had no energy, but she was still able to hold on to her job. She had no history of hepatitis, tuberculosis and other infectious diseases; no history of cholangitis, gallstones and other diseases; she was not taking any other medication and had no history of drug or food allergy. There is no history of surgical trauma or blood transfusion. She was born in Hangzhou, studied locally since childhood, and came to Sichuan after graduating from Zhejiang University 12 years ago. She worked at the Biological Institute of the Provincial Branch of Science and Technology, where she was heavily involved in scientific research and often stayed up late at night and travelled on business. No history of toxic exposure in the laboratory. Smoking for 10 years, 10 cigarettes/day. Does not drink alcohol. Married for 10 years. Lover 36 years old, healthy, occasional insomnia, working in the same unit, living in the unit dormitory, living conditions are good. Daughter 9 years old, healthy, no special diseases. Father 66 years old, suffered from hypotension for 2 years, ZMC outpatient treatment; mother 60 years old, suffered from coronary heart disease, also in ZMC outpatient treatment. |
| Okay, I will play the role of the patient, Zhang Hong, and you can start asking questions about the condition in order to make a diagnosis. You can ask me any questions about my condition, lifestyle habits, family history, etc. Please start asking questions. |  |
|  | You need to answer the questions I'm asking you based on the facts. Please perform like an ordinary person that does not have much professional knowledge in the medical field. Avoid using professional jargon. When I present a summary of symptoms and seek your confirmation, please first respond to my inquiry. Subsequently, simulate a patient tone characterized by anxiety, posing questions like 'Can my illness be cured?' or 'Is this a serious or minor illness?'  Just answer the question each time I gave you, do not provide information that is not related to my questions.  Please follow strictly with the information in the medical record I provided you, do not compile information that is already provided in medical record.  When giving a score, please only focus on the conversation happened in standardized patient simulation, for each list in criteria, review our conversation history to check if I have ever asked this question, if not, then you give me a zero for this list.  The patient’s all disease history and symptoms are as described in medical record. The patient does not have any disease or symptoms not mentioned.  At the end of my consultation, you need to rate my consultation process. |
| Understood. Please start asking questions. I will follow these rules to the letter. |  |
|  | Can you tell me exactly where you are feeling the pain? Can you describe the nature of the pain in detail? |
| The pain is mainly located in the upper abdomen, just below the raphe, and feels like a burning or dull ache. |  |
|  | Is your pain constant, or is it intermittent? |
| It is intermittent pain. It usually occurs after meals or at night and each episode lasts for about half a month before it gets better. |  |
|  | Under what conditions does your pain get better? |
| The pain is reduced after eating or drinking hot drinks. In addition, rest and dietary moderation (e.g., eating soft and light foods) may make symptoms better. |  |
|  | Has there been any recent change in your condition? |
| Yes, it has worsened in the last 3 months. I have been staying up late a lot and living irregularly, and the epigastric pain has flared up again with nausea and vomiting of coeliacs on two occasions, with the vomit smelling of hangover, in a relatively large amount. Symptoms persist despite improvement with treatment. |  |
|  | Well, it's possible that this could be a trigger for the onset of the disease. |
| Yes, I have a stressful job and often stay up late, which could be a trigger for the aggravation. |  |
|  | I see. When did the pain first appear? |
| The pain first appeared about 4 years ago. At that time, I started to feel pain in my upper abdomen due to irregular diet and stress at work. |  |
|  | Are your main symptoms only abdominal pain? |
| In addition to the epigastric pain, I had other symptoms such as nausea, occasional vomiting, and a 5kg weight loss. Appetite was also reduced, from a daily intake of 400 grams of food to 200 grams. In addition, I felt weak but was able to continue working. |  |
|  | Okay, I got it. Do you have any radiating pain, please? |
| Yes, the pain sometimes involves the back. |  |
|  | Have you recently lost any weight? If so, by how many kilograms? |
| Yes, I have lost 5kg in the last 4 years. |  |
|  | Do you have any symptoms of acid reflux? |
| I have no symptoms of acid reflux as of yet. |  |
|  | Do you have any belching symptoms? |
| No, no symptoms of belching. |  |
|  | OK, what about vomiting, have you ever vomited blood? |
| No symptoms of vomiting blood. |  |
|  | That’s good to hear, do you have any black stools? |
| There have been no instances of black stools. |  |
|  | OK, I have an overall understanding of your symptoms right now, let’s talk about your basic situation. Do you usually feel lethargic? |
| Yes, I did feel physically weak but managed to keep working. |  |
|  | Don’t push yourself too hard! How's your appetite? |
| I have lost my appetite. I used to be able to eat about 400 grams of food a day, but now it's down to about 200 grams. |  |
|  | When was your first visit? Were there any problems found at that time? |
| I was in the hospital two years ago and at that time the doctor recommended me to have a gastroscopy but I did not undergo it. I took Gastrozine and my symptoms improved after about 6 to 7 days and then stopped taking it on my own after a month. |  |
|  | I see, how did you feel after your first treatment? |
| The first treatment worked pretty well. My symptoms improved within about a week after taking GastroSupplement and I stopped taking the medication on my own after a month. At that time I felt a significant improvement in my condition. |  |
|  | Is this all for what happened in the first treatment? |
| Yes, the first treatment was all that. I just took GastroSuprine, no other treatments and no gastroscopy. After taking GastroSuprine, the symptoms improved. |  |
|  | Okay, let me review your situation. Your main symptom is epigastric pain for 4 years, aggravated for 3 months. The pain occurs when you are tired or have an irregular diet, it is burning or dull pain, relieved after eating, night pain, pain after meals, no acid reflux, no warmth, and nausea and vomiting for the past 3 months. She has had nausea and vomiting for the past 3 months, and was seen in the hospital 2 years ago, and was relieved by taking GastroSuprine. |
| Yes, your summary is correct. My main symptom is epigastric pain which has lasted for 4 years and worsened in the last 3 months. The pain occurs mostly after exertion or irregular eating and manifests itself as a burning or dull ache that is relieved by eating. I have night pain and pain after meals but no acid reflux or belching. It has also been accompanied by nausea and vomiting for the last 3 months. After seeing a doctor two years ago, taking gastroparesis relieves the symptoms.  (Mimics patient's anxious tone) May I ask if these symptoms are serious? Can I be cured? |  |
|  | Please be rest assured, there is no big deal. Now that the consultation is over, please rate my consultation process based on the 35 scoring criteria I am about to send out, and when giving your score, please focus only on the dialogue in the standardized patient simulation, and for each item in the scoring criteria, look back at the history of our conversations and check if I have ever asked the question before, and if I have not, then give me a zero on that item. Please note that each item is scored out of one point, if it has been asked that is one point, if not it is zero points |
| Please send in your 35 rating criteria and I will rate your consultation process based on those criteria. |  |
|  | The specific scoring rules are as follows: 1. Main symptoms 2. Time of appearance 3. Changes in condition 4. Triggering factors for the onset of the disease 5. Site of abdominal pain 6. Nature of abdominal pain 7. Degree of abdominal pain 8. Time and regularity of attacks 9. Relieving factors for abdominal pain 10. Whether there is radiating pain 11. Acid reflux or not 12. Whether belching or not 13. Whether nausea or vomiting or not 14. Whether vomiting or not 15. Whether black stools or not 16. Whether there is weight loss or not 17. First medical consultation 18. First Treatment 19. Effect of the first treatment 20. Second visit 21. Second treatment 22. Effect of the second treatment 23. Poor appetite, only half of the original amount of food 24. Weight loss of 5kg 25. Weakness 26. History of disease 27. History of trauma and surgery 28. History of food or drug allergy 29. Occupation 30. History of smoking 31. History of drinking 32. Marital history 33. Family members' health status 34. History of similar diseases 35. Health of family members 34, Any similar diseases 35, Any hereditary diseases |
| Based on the scoring criteria you provided, I will score based on our dialogue history:  1. **Main symptom** - 1 point (asked about epigastric pain)  2. **Time of presentation** - 1 point (asked about when the pain first appeared)  3. **Change in condition** - 1 point (asked about recent changes in condition)  4. **Trigger of onset** - 1 point (mentioned possible triggers)  5. **Site of abdominal pain** - 1 point (asked about the exact location of the pain)  6. **Nature of abdominal pain** - 1 mark (asked about the nature of the pain)  7. **Degree of abdominal pain** - 0 points (did not ask about the degree of pain)  8. **Time and pattern of episodes** - 1 point (asked about the time and pattern of pain episodes)  9. **Factors relieving abdominal pain** - 1 point (asked about factors relieving pain)  10. **Any radiating pain** - 1 point (asked about the presence of radiating pain)  11. **Any acid reflux** - 1 point (asked about acid reflux)  12. **Any belching** - 1 point (asked if belching was present)  13. **Any nausea and vomiting** - 1 point (asked if nausea and vomiting were present)  14. **Whether there was vomiting of blood** - 1 point (asked if there was vomiting of blood)  15. **Any black stool** - 1 point (asked if there was black stool)  16. **Any weight loss** - 1 point (asked if weight loss)  17. **First visit** - 1 point (asked about first visit)  18. **First treatment** - 1 point (asked about first treatment)  19. **Effect of first treatment** - 1 point (asked about the effect of the first treatment)  20. **Second visit** - 0 points (no mention of second visit)  21. **Second treatment** - 0 points (did not mention the second treatment)  22. **Effectiveness of second treatment** - 0 points (no mention of the effectiveness of the second treatment)  23. **Poor appetite and half of the original amount of food** - 1 point (enquired about the change in appetite and amount of food)  24. **Weight loss 5kg** - 1 point (asked about weight loss)  25. **Weakness** - 1 point (inquired about feeling weak)  26. **History of illness** - 0 points (no history of illness mentioned)  27. **History of traumatic surgery** - 0 points (history of traumatic surgery not mentioned)  28. **History of food or drug allergy** - 0 points (no history of food or drug allergy mentioned)  29. **Occupation** - 0 points (no occupation mentioned)  30. **Smoking history** - 0 points (no smoking history mentioned)  31. **Alcohol consumption history** - 0 points (no alcohol consumption history mentioned)  32. **Marriage history** - 0 points (no marriage history mentioned)  33. **Health of family members** - 0 points (health of family members not mentioned)  34. **Any similar illness** - 0 points (no mention of similar illness)  35. **Any hereditary diseases** - 0 points (no mention of hereditary diseases)  ***Total score: 21 out of 35.***  Please let me know if you have any additional questions or need further clarification. |  |
